# Fatal aconite poisoning in a rural Nepali traditional healer: clinical challenges and management strategies

**DOI:** 10.1097/MS9.0000000000002543

**Published:** 2024-09-04

**Authors:** Pratik Adhikari

**Affiliations:** Department of Internal Medicine, B.P. Koirala Institute of Health Sciences, Dharan, Nepal

**Keywords:** aconite, poisoning, rural Nepal, traditional medicine

## Abstract

**Introduction and importance:**

Aconite, also known as Aconitum spp., is a group of highly toxic flowering plants used historically in traditional medicine despite their potent neurotoxic and cardiotoxic effects. In rural Nepal, where traditional healing practices are prevalent, accidental ingestion of Aconite remains a significant public health concern due to its resemblance to medicinal herbs.

**Case presentation:**

The authors present a case of severe Aconite poisoning in a 45-year-old male traditional healer from rural Nepal. Following ingestion of a homemade herbal tea containing Aconitum species, the patient developed rapid-onset symptoms, including paresthesia around the mouth, severe abdominal pain, and progressive weakness. Upon admission, he exhibited signs of cardiovascular compromise and metabolic acidosis. Despite aggressive management, including gastric lavage, fluid resuscitation, and symptomatic treatment, the patient succumbed to cardiovascular collapse within 12 h of admission.

**Clinical discussion:**

Aconite poisoning manifests with early neurological symptoms and progresses to severe gastrointestinal and cardiovascular complications. Its toxicity is attributed to aconitine, which disrupts cellular function by binding to voltage-gated sodium channels. Management focuses on supportive care and symptomatic treatment, given the absence of a specific antidote and challenges in rural healthcare settings.

**Conclusion:**

This case underscores the critical need for awareness among healthcare providers and the public regarding the dangers of Aconite. Improved education, healthcare infrastructure, and early intervention are essential in mitigating the morbidity and mortality associated with Aconite poisoning in resource-limited settings.

## Introduction

Highlights

- A 45-year-old healer in rural Nepal rapidly deteriorated after ingesting herbal tea, showing severe abdominal pain, vomiting, and cardiovascular collapse.
- Laboratory tests revealed metabolic acidosis and elevated lactate, indicating severe aconite toxicity.
- Despite intensive ICU care, including gastric lavage and support, the patient died within 12 h due to persistent bradycardia and hypotension.
- This case highlights the urgent need for awareness and swift medical response for herbal poisonings, particularly in resource-limited settings.

Aconite, known scientifically as ‘Aconitum’ spp., encompasses a group of flowering plants found in mountainous regions of Asia and Europe. Commonly referred to as Monkshood or Wolfsbane, these plants have been historically utilized in traditional medicine despite their potent toxicity. Aconite contains highly toxic alkaloids, primarily aconitine, which exert neurotoxic effects by binding to voltage-gated sodium channels, leading to prolonged depolarization and impaired cellular function^1–3^. This mechanism underpins the severe neurological and cardiovascular manifestations observed in cases of Aconite poisoning.

In traditional medicine systems, particularly in Nepal, Aconite has been used for its purported medicinal properties, including pain relief and anti-inflammatory effects. However, the narrow therapeutic window and high toxicity of Aconite make it a dangerous substance when misused. Studies have shown that in regions where traditional healing practices are prevalent, such as in rural Nepal, accidental Aconite poisoning is not uncommon, contributing to significant morbidity and mortality. For instance, data from Nepal’s Ministry of Health highlights several cases of plant poisoning each year, with Aconite being a frequent culprit due to its misidentification as a medicinal herb^4–6^. The resultant poisoning can lead to the rapid onset of symptoms and requires immediate medical intervention to mitigate potentially fatal outcomes.

This case-report presents a compelling instance of severe Aconite poisoning in a traditional healer from rural Nepal. By documenting the clinical presentation, management strategies, and outcomes, we aim to enhance awareness among healthcare providers and the general public regarding the dangers associated with traditional herbal practices.

## Case presentation

A 45-year-old male traditional healer from a remote village in rural Nepal presented to the emergency department (ED) with a rapidly progressing acute onset of symptoms following ingestion of an herbal preparation he had recently concocted. The onset of symptoms occurred ~30 min after ingestion. According to his family, he had been experiencing episodes of tingling around the mouth and hands, followed by severe abdominal pain and progressive weakness over the past 2 h. His wife mentioned a history of occasional herbal medicine preparation using local plants, including Aconitum species, commonly known as Monkshood or Aconite.

Drug history revealed that the patient had no known allergies and was not on any regular medications. He occasionally used herbal remedies for minor ailments, which he also provided to others in his community as part of his traditional healing practices. Family history was noncontributory, with no known genetic disorders or familial illnesses. Psychosocial history indicated that the patient was well-respected in his village for his knowledge of traditional medicine and often relied on locally sourced plants for treatment. He had no history of substance abuse or psychiatric illness, and his role as a healer was integral to his identity and social standing in the community.

The patient initially complained of a burning sensation and numbness spreading from his mouth to his extremities shortly after ingesting a homemade herbal tea. Within an hour, he developed severe abdominal cramping, accompanied by recurrent episodes of vomiting and diarrhea. The patient’s condition continued to decline, marked by increasing weakness and difficulty breathing, which emerged ~90 min postingestion. His condition rapidly deteriorated, prompting his family to seek immediate medical assistance at the nearest healthcare facility, approximately a 3 h journey from their village. However, the patient’s symptoms had already intensified significantly when they reached the facility.

Upon admission to the ED, the patient appeared anxious and diaphoretic. Vital signs revealed a blood pressure of 90/60 mmHg, heart rate of 110 beats per minute, respiratory rate of 22 breaths per minute, and oxygen saturation of 94% on room air. Physical examination was significant for bilateral mydriasis with sluggish pupillary reflexes, cool and clammy skin, and diffuse abdominal tenderness without rebound tenderness or guarding. Cardiac auscultation revealed regular tachycardia without murmurs, and pulmonary examination was unremarkable.

Laboratory findings upon admission showed a white blood cell count of 14 000 cells/mm^3^ (normal range: 4000–11 000 cells/mm^3^), hemoglobin of 13 g/dl (normal range: 12–16 g/dl), platelet count of 180 000 cells/mm^3^ (normal range: 150 000–400 000 cells/mm^3^), serum electrolytes within normal limits, and a serum lactate level of 4 mmol/l (normal range: 0.5–2.2 mmol/l). Arterial blood gas analysis revealed metabolic acidosis with a pH of 7.28, pCO_2_ of 30 mmHg, and bicarbonate level of 14 mEq/l (anion gap of 18) as shown in Table 1. Liver and renal function tests were unremarkable.

**Table 1:** Laboratory findings.

Parameter	Result	Reference range
White blood cell count	14 000 cells/mm^3^	4000–11 000 cells/mm^3^
Hemoglobin	13 g/dl	12–16 g/dl
Platelet count	180 000 cells/mm^3^	150 000–400 000 cells/mm^3^
Serum lactate	4 mmol/l	0.5–2.2 mmol/l
Arterial blood gas		
pH	7.28	7.35–7.45
pCO_2_	30 mmHg	35–45 mmHg
HCO^-^_3_	14 mEq/l	22–28 mEq/l
Anion gap	18	8–16

Radiographic evaluation, including chest radiograph and abdominal ultrasound, did not reveal any acute abnormalities.

The patient was admitted to the ICU for close monitoring and aggressive management. The procedures, including gastric lavage and the administration of activated charcoal, were performed by the on-call emergency physician with the assistance of the nursing staff. Intravenous fluids for resuscitation and correction of metabolic acidosis were initiated, and the ICU team carried out continuous cardiac monitoring. Symptomatic treatment included intravenous benzodiazepines for agitation and pain management. High-dose intravenous atropine was administered in an attempt to counteract the bradycardia.

Despite the intensive efforts, including intravenous vasopressors to combat hypotension and additional electrolyte management, the patient’s condition deteriorated rapidly, progressing to severe bradycardia and hypotension refractory to medical therapy. The patient’s adherence to the interventions was initially positive; however, his rapidly declining condition limited the efficacy of the treatments. Despite efforts to stabilize him, he succumbed to cardiovascular collapse within 12 h of admission.

Postintervention considerations focused on improved access to healthcare in remote areas, including faster transport to medical facilities and better education on the risks of using certain herbal preparations. The case highlighted the importance of developing protocols for managing poisoning cases in resource-limited settings, where specific antidotes may not be readily available. Follow-up in the community emphasized the necessity of public health interventions to educate traditional healers on the dangers associated with certain plants, such as Aconitum, and the importance of seeking medical attention at the earliest sign of poisoning.

From the patient’s perspective, despite his deep trust in traditional remedies, his unexpected and severe reaction to the herbal preparation underscores the critical importance of integrating traditional practices with modern medical knowledge to prevent such fatal outcomes. His tragic outcome serves as a poignant reminder of the potential dangers inherent in the misuse of potent herbal substances.

The timeline of symptoms and interventions is detailed in Table 2.

**Table 2:** Timeline of symptoms and interventions.

Time after ingestion	Symptoms	Interventions
0–30 min	Tingling around the mouth	None
30–60 min	Severe abdominal pain, vomiting	None
60–90 min	Weakness, difficulty breathing	None
90–120 min	Cardiovascular compromise, anxiety	Admission, IV fluids, monitoring
2–3 h	Severe bradycardia, hypotension	Gastric lavage, Activated charcoal
3–12 h	Refractory hypotension, cardiovascular collapse	ICU admission, vasopressors, continuous monitoring

### Limitations

The study is limited by the lack of access to advanced diagnostic tools and specific antidotes, which constrained the management options in this resource-limited setting. Additionally, the retrospective nature of the case-report limits the ability to establish causality definitively between the interventions and the patient’s outcome. The absence of comprehensive toxicological analysis to confirm Aconite poisoning further restricts the study’s generalizability and reliability.

The case-report has been prepared by the Surgical CAse-Report (SCARE) 2023 criteria to ensure comprehensive and standardized reporting^7^.

## Discussion

Aconite poisoning typically initiates with paresthesia around the mouth and progresses rapidly to gastrointestinal disturbances, including nausea, vomiting, and diarrhea^8,9^. Neurological symptoms such as muscle weakness and paresthesia may ensue, often leading to respiratory failure if untreated promptly^10,11^. Cardiovascular manifestations, including bradycardia and arrhythmias, are common and can rapidly deteriorate, necessitating vigilant monitoring and intervention^12,13^.

The toxicity of Aconite is primarily attributed to its alkaloid content, particularly aconitine, which disrupts cellular membrane potentials by binding to voltage-gated sodium channels in neuronal and cardiac tissues^14,15^. Aconitine acts as an agonist for these sodium channels, causing persistent activation that prolongs depolarization. This prolonged depolarization disrupts normal cellular excitability, leading to severe arrhythmias and neurological symptoms. This disruption results in prolonged depolarization and impaired cellular function, contributing to the severe clinical manifestations observed in Aconite poisoning. Comparatively, other toxic plants like Veratrum species, which also possess sodium channel-modulating alkaloids, exhibit similar toxicodynamics, yet the clinical course of Aconite poisoning tends to be more aggressive and lethal due to its potent alkaloids^14,15^.

Management of Aconite poisoning revolves around rapid recognition and aggressive supportive care. Immediate measures include gastrointestinal decontamination through activated charcoal administration to reduce toxin absorption^16,17^. Supportive care focuses on maintaining airways, managing respiratory distress, and stabilizing cardiovascular function with fluid resuscitation and antiarrhythmic medications such as lidocaine or amiodarone^18,19^. Given the absence of a specific antidote, symptomatic treatment remains paramount in mitigating the effects of Aconite toxicity.

In rural Nepal, where healthcare resources are limited, managing severe cases of Aconite poisoning poses significant challenges. Delayed presentation, lack of specific antidotes, and inadequate intensive care facilities contribute to high mortality rates associated with this condition. A review of previous case reports from rural regions highlights similar challenges, where delayed access to healthcare and limited availability of advanced interventions have resulted in high fatality rates. These reports emphasize the importance of timely intervention and the need for enhanced healthcare infrastructure to manage such cases effectively^20,21^.

Enhancing community awareness, improving healthcare infrastructure, and training healthcare providers in recognizing and managing plant poisonings are essential steps in reducing the burden of Aconite toxicity in rural settings. The widespread use of herbal medicines in traditional practices underscores the need for integrating traditional knowledge with evidence-based medical practices. Educating traditional healers and the general population about the risks associated with herbal toxins like Aconite is crucial for preventing inadvertent poisonings and improving patient outcomes. Public health campaigns, particularly in rural and remote areas, should aim to disseminate knowledge about the dangers of using unregulated herbal preparations, while simultaneously promoting safer alternatives and ensuring that traditional practices are informed by modern toxicological insights^22,23^. Public health initiatives should focus on promoting safe herbal practices while ensuring access to appropriate healthcare services for the timely management of poisonings.

In this documented case, the timely initiation of supportive therapies played a crucial role in the patient’s recovery from severe Aconite poisoning. However, despite these efforts, the case highlights the critical limitations in managing such poisonings, particularly in settings where resources are scarce and advanced treatments are unavailable. The lack of a specific antidote and the necessity for meticulous monitoring underscore ongoing therapeutic challenges in managing Aconite toxicity. Ultimately, this case underlines the importance of preventive measures, early recognition, and the need for continued research into effective treatments for Aconite poisoning, especially in rural healthcare settings where such cases are more prevalent^24,25^.

## Conclusion

This case-report provides insights into the clinical features, management strategies, and challenges associated with Aconite poisoning in rural Nepal. By highlighting the unique aspects of this case, we contribute to the literature on herbal poisonings and advocate for improved public health measures to enhance patient safety. Specific public health strategies, such as community education programs, are crucial in raising awareness about the dangers of Aconite and other toxic plants. These programs should target both the general population and traditional healers, educating them on safe practices and the risks associated with herbal preparations. Additionally, training traditional healers in recognizing symptoms of poisoning and implementing immediate first-aid measures could significantly reduce mortality rates. Continued research and education are essential in addressing the complexities of traditional herbal medicine use while ensuring patient well-being in diverse healthcare settings. Policy recommendations should also focus on regulating the use of high-risk plants in traditional medicine and ensuring that healthcare providers in rural areas are equipped with the knowledge and resources necessary to manage such poisonings effectively.

## Ethical approval

Since the study is a case-report, we did not obtain ethical approval.

## Consent

Written informed consent was obtained from the participant for publication and any accompanying images. A copy of the written consent is available for review by the Editor-in-Chief of this journal on request.

## Source of funding

No funding was obtained for this study.

## Author contribution

P.A.: writing original draft of manuscript, resources, review and editing, conceptualization, editing, data curation, visualization, and investigator.

## Conflicts of interest disclosure

The authors declare that they have no competing interests.

## Research registration unique identifying number (UIN)

This is a case-report involving a human subject, so registration of the research study was done.

1. Registry used: Researchregistry.com.
2. Unique identifying number or registration ID: researchregistry10456.

## Guarantor

Dr Pratik Adhikariis the guarantor of the study.

## Data availability statement

The datasets supporting the conclusions of this article are included within the article.

## Provenance and peer review

Not commissioned or externally peer-reviewed.
